# Molecular Pathways Involved in Prostate Carcinogenesis: Insights from Public Microarray Datasets

**DOI:** 10.1371/journal.pone.0049831

**Published:** 2012-11-20

**Authors:** Sarah C. Baetke, Michiel E. Adriaens, Renaud Seigneuric, Chris T. Evelo, Lars M. T. Eijssen

**Affiliations:** 1 Department of Bioinformatics - BiGCaT, Maastricht University, Maastricht, The Netherlands; 2 Department of Experimental Molecular Imaging, Helmholtz Institute for Biomedical Engineering, RWTH Aachen University, Aachen, Germany; 3 Department of Experimental Cardiology, Heart Failure Research Center, Academic Medical Center, University of Amsterdam, Amsterdam, The Netherlands; 4 Université de Bourgogne, Dijon, France; 5 Heat Shock Proteins and Cancer, INSERM, UMR 866 IFR 100, Faculty of Medicine and Pharmacy, Dijon, France; Ghent University, Belgium

## Abstract

**Background:**

Prostate cancer is currently the most frequently diagnosed malignancy in men and the second leading cause of cancer-related deaths in industrialized countries. Worldwide, an increase in prostate cancer incidence is expected due to an increased life-expectancy, aging of the population and improved diagnosis. Although the specific underlying mechanisms of prostate carcinogenesis remain unknown, prostate cancer is thought to result from a combination of genetic and environmental factors altering key cellular processes. To elucidate these complex interactions and to contribute to the understanding of prostate cancer progression and metastasis, analysis of large scale gene expression studies using bioinformatics approaches is used to decipher regulation of core processes.

**Methodology/Principal Findings:**

In this study, a standardized quality control procedure and statistical analysis (http://www.arrayanalysis.org/) were applied to multiple prostate cancer datasets retrieved from the ArrayExpress data repository and pathway analysis using PathVisio (http://www.pathvisio.org/) was performed. The results led to the identification of three core biological processes that are strongly affected during prostate carcinogenesis: cholesterol biosynthesis, the process of epithelial-to-mesenchymal transition and an increased metabolic activity.

**Conclusions:**

This study illustrates how a standardized bioinformatics evaluation of existing microarray data and subsequent pathway analysis can quickly and cost-effectively provide essential information about important molecular pathways and cellular processes involved in prostate cancer development and disease progression. The presented results may assist in biomarker profiling and the development of novel treatment approaches.

## Introduction

Prostate cancer is currently the most frequently diagnosed malignancy in men and the second leading cause of cancer-related morbidity and mortality in industrialized countries [1]–[3]. Worldwide, more than 650,000 new cases of prostate cancer are diagnosed each year, accounting for 10% of all new male cancer cases [4]. Furthermore, it is estimated that the incidence of prostate cancer will even rise due to an increased life-expectancy, aging of the population and improved and earlier detection [1], [4].

Although the specific underlying mechanisms of prostate carcinogenesis have not been unraveled yet, it is supposed that prostate cancer results from a combination of genetic and environmental factors, including several susceptibility genes for inherited prostate cancer, ethnicity and family history, as well as different dietary and life style factors [1], [3], [5]–[7].

Due to the complex etiology of prostate cancer, treatment options for prostate cancer patients depend on multiple factors, including a patient’s age and general health status, the prostate specific antigen (PSA) level, as well as the tumor grade and status. One treatment option for localized prostate cancer is radical prostatectomy, the surgical removal of the prostate gland and nearby lymph nodes. However, it is estimated that 25–40% of men undergoing radical prostatectomy will have disease relapse, as detected by increasing serum levels of PSA [8]. Another treatment option for prostate cancer is androgen ablation therapy that has become the standard treatment in advanced cases of prostate cancer. It prevents testosterone production by the testes leading to prostate cancer cell depletion and subsequent tumor regression in the short-term. Androgen deprivation is either achieved by surgical or chemical castration, which can be performed by the administration of estrogens and gonadotropin-releasing agonists and antagonists, and has been shown to be effective in the treatment of advanced diseases. However, androgen depletion is often associated with disease recurrence, as indicated by elevated PSA levels. This recurrent form of prostate cancer is known as androgen-independent, an essentially untreatable form of prostate cancer that ultimately progresses and metastasizes. In this aggressive type of prostate cancer, the administration of the most effective standard chemotherapeutic regimens only leads to a mean increase in survival time of two months [2], [3]. Therefore, a major challenge in scientific research will be the elucidation of the underlying mechanisms of androgen-independent prostate cancer. Also deciphering the molecular networks that distinguish progressive from non-progressive disease will shed light on the biology of metastasizing prostate cancer and will ultimately lead to the identification of novel biomarkers and treatment strategies.

Gene expression microarray technology has been the method of choice for monitoring the complex expression patterns between the numerous molecular players such as those involved in prostate cancer. Bioinformatics tools, including quality control (QC) and analysis of the generated data up to the biological pathway level, are required to identify key genes and cellular pathways involved in prostate cancer development and progression.

**Figure 1:**
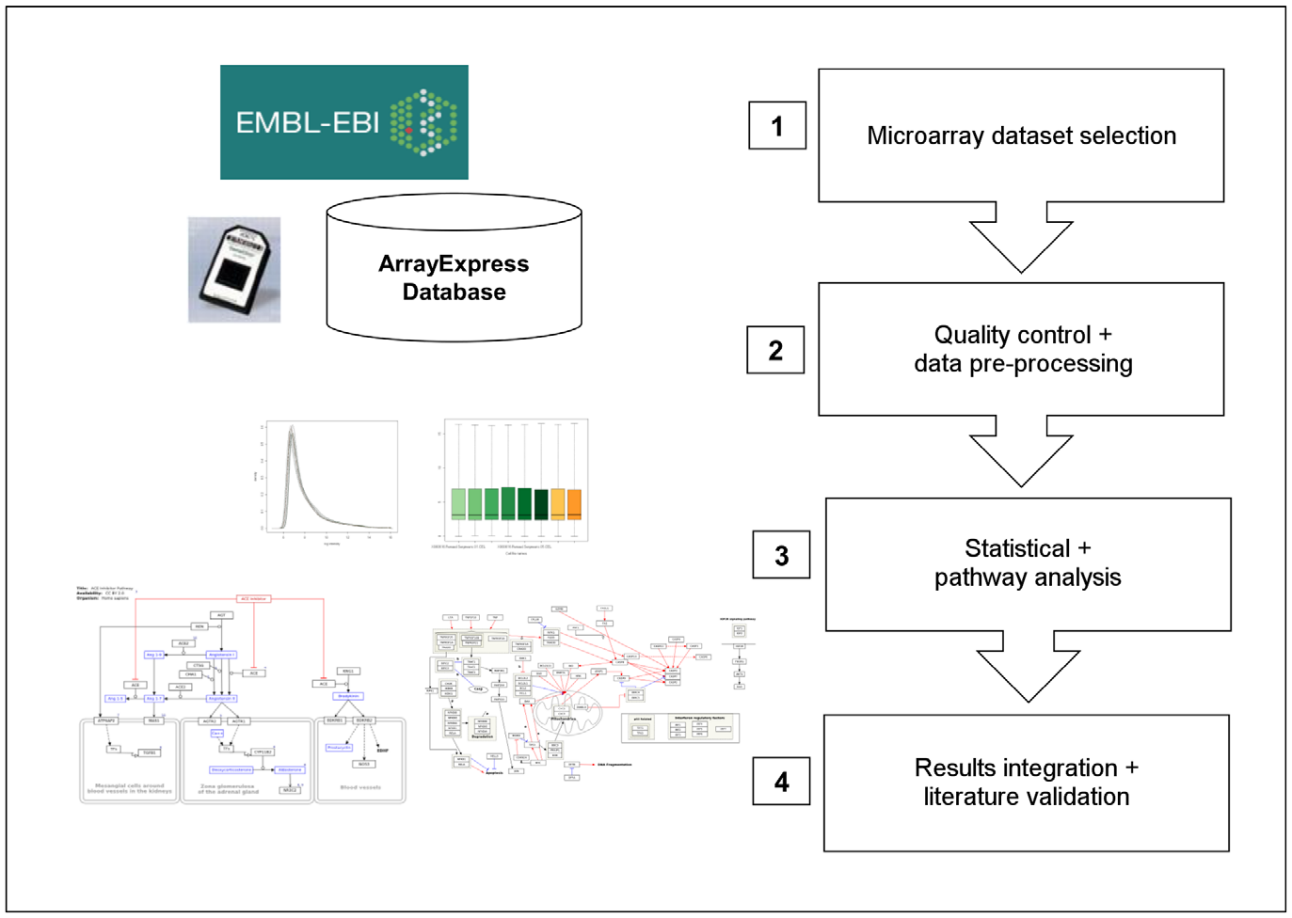
Standardized microarray data analysis workflow. Starting from the publicly available EMBL repository ArrayExpress: 1) Relevant prostate cancer studies were selected and downloaded; 2) Quality control and data pre-processing steps were performed in the R environment. Microarrays with insufficient sample quality, hybridization quality, signal comparability or array correlation were excluded; 3) For each included study, statistical analysis was performed and pathway analysis was run with PathVisio to identify the biological processes involved; 4) Results were then integrated and compared to literature findings.

This study involves microarray data analysis using the open source language R [9], applying QC and analysis tools by running a standardized workflow developed at the BiGCaT department (http://www.arrayanalysis.org/) to multiple datasets. An overview of the workflow is depicted in Figure 1.

Datasets are selected from the public repository ArrayExpress (http://www.ebi.ac.uk/arrayexpress/) based on their relevance to ongoing cancer research focusing on prostate cancer [10]. Standardized QC, pre-processing, statistical analysis, and subsequent pathway analysis using PathVisio with WikiPathways content [11], [12] were applied to robustly identify key genes and biological processes playing an important role in prostate cancer. Ultimately, the adaptation of standardized procedures to multiple datasets will ease and speed up data QC and analysis and as such enhance biomarker profiling and accelerate the retrieval of novel therapeutic targets in prostate cancer and other diseases. Joint evaluation of existing relevant datasets is pivotal to efficient and cost effective systems biology research, here illustrated by extracting and validating the core processes involved in prostate cancer etiology and progression.

## Materials and Methods

### Microarray Dataset Selection

We searched the open repository ArrayExpress (http://www.ebi.ac.uk/arrayexpress/) of the European Bioinformatics Institute for datasets meeting the following inclusion criteria: (i) investigating human prostate cancer; (ii) performed on the Affymetrix GeneChip platform; (iii) providing raw data CEL files as well as processed files. Based on these selection criteria, five different datasets were selected. An overview of the characteristics of these datasets is given in Table 1.

**Table 1:** Characteristics of the selected datasets.

Dataset	ArrayExpress ID	Array type	Number of samples
**Varambally ** ***et al.***	E-GEOD-3325	Affymetrix Human Genome U133 Plus 2.0	19 (NP: 6, pPC: 7, mPC: 6) (−0)
**Wallace ** ***et al.***	E-GEOD-6956	Affymetrix Human Genome U133A 2.0	46 (NP: 11 (−3), PC: 35 (−1)
**Sun ** ***et al.***	E-GEOD-25136	Affymetrix Human Genome U133A	79 (indolent: 40 (−2), recurrent: 39 (−4))
**Best ** ***et al.***	E-GEOD-2443	Affymetrix Human Genome U133A	20 (AI: 10 (−1), AD: 10 (−1))
**Gregg ** ***et al.***	E-GEOD-20758	Affymetrix Human Genome U133 2.0	6 (PC: 3, stromal cell samples: 3) (−6)

The number of excluded arrays for each dataset using the standardized QC procedure is indicated in brackets. Abbreviations: NP: normal prostate, pPC: primary prostate cancer, mPC: metastatic prostate cancer, AI: androgen independent, AD: androgen dependent.

The first dataset by Varambally *et al.* (ArrayExpress ID: E-GEOD-3325) is composed of 19 Affymetrix Human Genome U133 Plus 2.0 arrays investigating six individual benign prostate tissue samples, seven primary prostate cancer samples, and six metastatic prostate cancer samples that were obtained from radical prostatectomy. The aim of the original study was to identify alterations in human prostate cancer and to reveal signatures of disease progression from clinically localized prostate cancer to metastatic prostate tumors [13].

The second dataset is a subset of 46 Affymetrix Human Genome U133A 2.0 GeneChips created from a gene expression experiment by Wallace *et al.* (E-GEOD-6956). The goal of the study by Wallace *et al.* was to perform a genome-wide gene expression profiling of prostate tumors to ultimately determine differences in tumor biology between African-American and European-American patients. As the effect of ethnicity was not a focus of our current study, only arrays concerning either normal prostate tissue (11 arrays) or adenocarcinoma samples (35 arrays) of European-American patients were selected [14].

The third dataset by Sun *et al.* (E-GEOD-25136) consists of 79 tissue samples obtained from patients with clinically localized prostate cancer treated by radical prostatectomy. Thirty-nine samples show disease recurrence as classified by three consecutive increases of PSA levels after radical prostatectomy. Forty samples are determined as non-recurrent samples based on undetectable serum PSA levels (<0.05 ng/ml) over a period of at least five years after radical prostatectomy. Gene expression analysis was performed using Affymetrix Human Genome U133A GeneChips. Furthermore, a computational analysis of the obtained gene expression profile data was conducted to examine whether advanced computational algorithms are able to derive more accurate prognostic signatures for prostate cancer [15].

The fourth dataset by Best *et al.* (E-GEOD-2443) consists of 20 Affymetrix Human Genome U133A GeneChips comparing the gene expression profiles of 10 androgen-dependent primary prostate tumor biopsies with 10 androgen-independent prostate cancer samples. The obtained expression profiles were originally analyzed with regard to metabolic pathways, gene ontologies and genomic alterations [16].

The fifth dataset by Gregg *et al.* (E-GEOD-20758) is composed of six Affymetrix Human Genome U133 2.0 GeneChips investigating cell-type specific gene expression patterns in prostate cancer. Prostatic adenocarcinoma epithelial cell samples and interstitial stromal cell samples of three individuals were obtained from laser capture microdissection (LCM) and their differential expression was analyzed. The identification of distinct gene expression patterns in prostate tumor epithelial cells and adjacent stromal cells was aimed to contribute to a better understanding of potential cellular interactions in prostate cancer [17].

### Microarray Data Analysis and Quality Control

Microarray data analysis was performed using the open source language R (version 2.13.0) and R packages of Bioconductor 2.8 [9], [18]. A variety of established array specific QC, visualization, normalization and statistical methods were combined into one workflow at the BiGCaT department (see http://www.arrayanalysis.org/) including GCRMA normalization and Ensembl ID based updated gene annotation from Brainarray (http://brainarray.mbni.med.umich.edu) [19]–[21].

Linear modeling using the limma package was conducted to compute the genes for each dataset that were significantly changed between experimental groups, as defined by a p-value smaller than 0.05 and these genes were mapped to biological pathways using PathVisio with WikiPathways content [11], [12], [21]. Using the statistics function in PathVisio, an ordered list of Z-score ranked pathways was generated based on the overrepresentation of significantly changed member genes between experimental groups. All pathways with a Z-score higher than 1.9 were included in the biological interpretation, resembling a significance level of 0.05.

Statistical and pathway analyses were applied to the data obtained by the standardized QC and normalization procedure (‘reprocessed data’) as well as to the normalized data as provided on ArrayExpress (‘published data’) for each dataset. The results of the reprocessed data and the published data were compared to get an overview of the overlap and differences in pathway analysis results. Results of all datasets were combined to robustly identify central biological pathways involved in prostate carcinogenesis. A detailed description of the applied methods and bioinformatics tools can be found in Appendix S1.

## Results

### Quality Control

**Figure 2:**
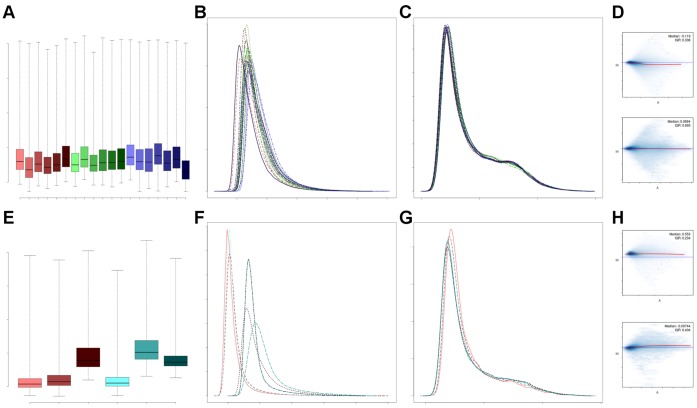
Overview of the QC results of two selected datasets. Several QC results of the dataset by Varambally *et al.* comparing arrays of benign prostate tissue (maroon), primary prostate cancer (blue) and metastatic prostate cancer samples (green) are shown in panel a-d. Several QC results of the dataset by Gregg *et al.* comparing arrays of prostatic epithelial (maroon) with interstitial stromal cell samples (teal) are depicted in panel e-h. a) Boxplot of raw intensities; b) density histogram of log intensities before normalization; c) density histogram of log intensities after normalization; d) MA-plot before (upper panel) and after normalization (lower panel) of one example array of the dataset by Varambally *et al.*; e) boxplot of raw intensities; f) density histogram of log intensities before normalization; g) density histogram of log intensities after normalization; h) MA-plot before (upper panel) and after normalization (lower panel) of one example array of the dataset by Gregg *et al.*

It is necessary to assess the quality of microarrays and select those having sufficient quality before running further analyses. To control for the quality of each microarray within a dataset, several metrics were computed, resulting in plots and bar diagrams as illustrated in Figure 2 with examples of two of the datasets. A link to the complete QC results can be found in Appendix S2.

The application of the standardized QC procedure to the dataset by Varambally *et al.* [13] revealed overall sufficient quality of the dataset. All indicators of sample quality, hybridization quality, signal comparability and array correlation met the QC criteria, so that no array needed to be excluded from the dataset. The boxplot of raw intensities (Figure 2a) and the density histogram of the log intensity distribution (Figure 2b) of each array before normalization provide an overview of dataset quality. Normalization was able to sufficiently remove smaller discrepancies, leading to comparable distributions between all arrays (Figure 2c). In Figure 2d, an MA-plot of one example array of the dataset by Varambally *et al.* before and after normalization is shown. MA-plots allow comparison of the log-intensity of each array to the dataset median and identification of intensity-dependent biases. The y-axis of the MA-plot shows the difference in logged intensity of one array to the reference median array, which is called M (minus). The x-axis indicates the average log-intensity of the arrays, which is called A (add). Assuming that the majority of genes are unchanged, the MA-plot should be spread symmetrically around the x-axis (y = 0). Furthermore, normalization is expected to correct for intensity-dependent biases.

QC of the dataset by Wallace *et al*. [14] indicated four arrays with an aberrant behavior. One array in the prostatic adenocarcinoma group and three arrays in the control group of normal prostate tissue did not fulfill the QC criteria and were removed from further analysis. After excluding the four indicated poor quality arrays and performing QC with the remaining arrays, data of sufficient quality were obtained.

QC of the dataset by Sun *et al.* [15] revealed six arrays of insufficient quality (two arrays in the non-recurrent and four arrays in the recurrent group) and two arrays (one array per group) with suspicion of low quality. In order to make sure that only data of sufficient quality meeting the QC criteria were kept for further analysis, all indicated arrays were removed and QC was performed again revealing overall sufficient quality of the remaining arrays.

QC of the dataset by Best *et al.* [16] clearly indicated two arrays of insufficient quality that did not meet the QC criteria and had to be excluded from further analysis. In each experimental group one array was removed, QC was performed again and data of sufficient quality fulfilling the QC criteria were obtained.

QC of the dataset by Gregg *et al*. [17] investigating cell-type specific gene expression patterns in prostate cancer revealed low quality of the whole dataset. The boxplot of raw intensities (Figure 2e) and density histogram of log intensities (Figure 2f) before normalization indicated strong differences between the arrays. The density histogram of log intensities after normalization is depicted in Figure 2g. The MA-plots before and after normalization of an example array selected from the dataset by Gregg *et al.* are shown in Figure 2h, indicating low quality of the array. Normalization was unable to correct for intensity-dependent biases and to center the MA-plot on the x-axis. Based on the limited number of arrays in the dataset and the fact that several arrays in both experimental groups did not fulfill the QC criteria, it was decided to exclude the entire dataset from further analysis.

### Pathway Analysis

Pathway analysis is considered to ease data interpretation and most importantly to lead to more robust results compared to only providing a signature of differentially expressed genes. Concordantly, it was expected that differences in the results of statistical analysis between the processed data using the standardized procedure and the processed data from ArrayExpress are mitigated by performing analysis at the level of biological pathways.

To this end, pathway analysis using PathVisio was performed to study changes at a biological process level, using pathway content from WikiPathways. Results were compared between the processed data obtained using the standardized procedure and the processed data downloaded from ArrayExpress. For the biological interpretation, only significant pathways with a Z-score higher than 1.9 in at least one of the comparisons were included.

Pathway analysis of the dataset by Varambally *et al.* [13] was performed based on the result of statistical analysis comparing differences in gene expression patterns between primary prostate cancer and benign prostate tissue and between metastatic and primary prostate cancer. The comparison between benign prostate tissue and primary prostate cancer revealed 16 biological pathways to be significantly changed and involved in the development of primary prostate cancer. These pathways and their calculated Z-scores are summarized in Table 2.

**Table 2:** PathVisio results of significant pathways found in the dataset by Varambally *et al.* comparing processed data provided by ArrayExpress with the reprocessed data.

Pathway	Z Score (ArrayExpress)	Z Score (Standardized processing)
*Cholesterol Biosynthesis*	***4.14***	***3.65***
Glutathione metabolism	**2.67**	1.59
Striated Muscle Contraction	**2.17**	0.78
Endochondral Ossification	**2.12**	1.32
Delta-Notch Signaling Pathway	**2.12**	0.68
*Hedgehog Signaling Pathway*	***2.10***	***2.53***
*Calcium Regulation in the Cardiac Cell*	***1.96***	***2.29***
Eicosanoid Synthesis	1.53	**1.94**
Prostaglandin Synthesis and Regulation	1.34	**2.51**
Id Signaling Pathway	1.14	**2.52**
Selenium	0.98	**2.84**
Nicotine Activity on Dopaminergic Neurons	0.69	**2.25**
Cytoplasmic Ribosomal Proteins	0.10	**2.48**
Irinotecan Pathway	0.07	**2.57**
Ganglio Sphingolipid Metabolism	−0.21	**2.47**
Sulfation	−0.34	**1.95**

Pathway analysis is based on a comparison between benign prostate tissue and primary prostate cancer. Only significant pathways with a Z-score >1.9 in at least one of the two analyses are included. Significant Z-scores are depicted in bold; matches in pathways between the analyses are in italics.

Pathway analysis of the published data from ArrayExpress detected 7 significantly altered pathways with a Z-score higher than 1.9, including e.g. the “Cholesterol Biosynthesis”, “Glutathione metabolism”, and “Delta-Notch Signaling Pathway”.

PathVisio results of the reprocessed data indicated 12 significantly changed pathways involved in prostate carcinogenesis, such as the “Cholesterol Biosynthesis”, “Hedgehog Signaling Pathway”, and “Selenium” pathway, amongst others. Three matches in significant pathways between the reprocessed data and the published data could be detected (Table 2), namely the “Cholesterol Biosynthesis”, “Hedgehog Signaling Pathway”, and the “Calcium Regulation in the Cardiac Cell” pathway. Except for the “Calcium Regulation in the Cardiac Cell” pathway, these pathways are expected to be involved in prostate cancer initiation, whereas the latter obviously is not involved in prostate carcinogenesis, but is composed of several sub-processes and regulators, such as the Na^+^/K^+^ATPase, the Na^+^/Ca^2+^exchanger, and regulators of G-protein signaling that might also be altered during prostate carcinogenesis.

Pathway analysis of the published data comparing metastatic with primary prostate cancer revealed 25 significantly changed pathways, while 20 significantly altered pathways in the reprocessed data could be detected. A summary of these pathway analysis results is given in Table 3.

**Table 3:** PathVisio results of significant pathways found in the dataset by Varambally *et al.* comparing processed data provided by ArrayExpress with the reprocessed data.

Pathway	Z Score (ArrayExpress)	Z Score (Standardized processing)
*Cell cycle*	***4.11***	***5.52***
*G1 to S cell cycle control*	***3.82***	***4.67***
Glucuronidation	**3.77**	0.62
*B Cell Receptor Signaling Pathway*	***3.65***	***2.21***
*Androgen Receptor Signaling Pathway*	***3.63***	***3.74***
*Signaling of Hepatocyte Growth Factor Receptor*	***3.51***	***2.48***
*T Cell Receptor Signaling Pathway*	***3.26***	***2.06***
EGFR1 Signaling Pathway	**2.94**	1.80
*Wnt Signaling Pathway*	***2.85***	***2.49***
*Apoptosis*	***2.83***	***3.38***
IL-3 Signaling Pathway	**2.81**	1.16
Wnt Signaling Pathway NetPath	**2.44**	1.55
IL-7 Signaling Pathway	**2.43**	1.40
FAS pathway and Stress induction of HSP regulation	**2.38**	0.48
*Delta-Notch Signaling Pathway*	***2.34***	***2.27***
DNA damage response (only ATM dependent)	**2.34**	1.56
*Toll-like receptor signaling pathway*	***2.28***	***1.93***
*TGF-beta Receptor Signaling Pathway*	***2.27***	***2.07***
p38 MAPK Signaling Pathway (BioCarta)	**2.23**	0.66
Senescence and Autophagy	**2.16**	1.72
*Wnt Signaling Pathway and Pluripotency*	***2.08***	***2.07***
Insulin Signaling	**2.06**	0.38
*DNA damage response*	***1.94***	***2.68***
*miRNAs involved in DDR*	***1.93***	***2.10***
Focal Adhesion	**1.91**	0.71
Endochondral Ossification	1.69	**2.49**
Nucleotide Metabolism	1.68	**2.29**
Myometrial Relaxation and Contraction Pathways	1.55	**2.24**
DNA Replication	1.27	**3.45**
One Carbon Metabolism	0.88	**2.41**
Angiogenesis	0.68	**1.90**

Pathway analysis is based on a comparison between primary prostate cancer and metastatic prostate cancer. Only significant pathways with a Z-score >1.9 in at least one of the two analyses are included. Significant Z-scores are depicted in bold; matches in pathways between the analyses are in italics.

A considerable overlap in pathways between the reprocessed data and the published data could be detected, where 14 complete matches in pathways were identified (Table 3). Several of those matches were found in pathways being expected to be involved in prostate cancer development and progression to metastatic disease, such as the “Cell cycle”, “G1 to S cell cycle control”, “DNA damage response”, and “Apoptosis” pathway. Other matches in pathways were indicated in the “Androgen Receptor Signaling Pathway”, and pathways involved in the immune response, like the “T Cell Receptor Signaling Pathway” and the “B Cell Receptor Signaling Pathway”.

Pathway analysis of the dataset by Wallace *et al.* [14] focusing on differences in gene expression between prostatic adenocarcinoma and benign prostate tissue identified 17 significantly altered pathways playing an essential role in prostate carcinogenesis. These pathways and their calculated Z-scores are summarized in Table 4.

**Table 4:** PathVisio results of significant pathways found in the dataset by Wallace *et al.* comparing processed data provided by ArrayExpress with the reprocessed data after quality control.

Pathway	Z Score (ArrayExpress)	Z Score (Standardized processing)
Cytoplasmic Ribosomal Proteins	**7.36**	0.17
*Electron Transport Chain*	***5.24***	***5.21***
EGFR1 Signaling Pathway	**4.64**	1.35
*mRNA processing*	***4.52***	***4.37***
TNF-alpha/NF-kB Signaling Pathway	**3.88**	1.05
*Oxidative phosphorylation*	***2.96***	***4.00***
Signaling of Hepatocyte Growth Factor Receptor	**2.94**	0.82
Androgen Receptor Signaling Pathway	**2.78**	0.57
*Translation Factors*	***2.48***	***2.06***
IL-9 Signaling Pathway	**2.44**	−0.34
T Cell Receptor Signaling Pathway	**2.36**	0.37
Non-homologous end joining	**2.35**	1.36
Proteasome Degradation	**2.25**	1.02
Serotonin Receptor 4/6/7 NR3C signaling	**2.23**	−0.49
Notch Signaling Pathway	**2.22**	−0.89
Fatty Acid Biosynthesis	**1.90**	−0.34
Insulin Signaling	1.49	**1.91**

Pathway analysis is based on a comparison between normal prostate tissue and prostatic adenocarcinoma. Only significant pathways with a Z-score >1.9 in at least one of the two analyses are included. Significant Z-scores are depicted in bold; matches between the analyses are in italics.

PathVisio analysis of the published data indicated 16 significantly altered pathways, like e.g. the “Cytoplasmic Ribosomal Proteins”, “Electron Transport Chain”, and the “EGFR1 Signaling Pathway” (Table 4). Pathway analysis of the reprocessed data after QC revealed 5 significant pathways. PathVisio results clearly identified four overlapping pathways that were found in the reprocessed data after QC, as well as in the published data. These pathways included the “mRNA processing”, “Electron Transport Chain”, “Oxidative phosphorylation” and ”Translation Factors” pathways (Table 4) and appeared to contribute to prostate carcinogenesis.

As depicted in Table 5, pathway analysis of the dataset by Sun *et al.* [15] comparing gene expression profiles of non-recurrent prostate cancer with disease relapse revealed 17 significantly altered pathways, but no match in pathways between the reprocessed data and the published data could be detected.

**Table 5:** PathVisio results of significant pathways found in the dataset by Sun *et al.* comparing processed data provided by ArrayExpress with the reprocessed data after quality control.

Pathway	Z Score (ArrayExpress)	Z Score (Standardized processing)
miRNAs involved in DDR	**3.30**	−1.22
Angiogenesis	**2.42**	0.22
IL-2 Signaling Pathway	**2.39**	−0.94
FAS pathway and Stress induction of HSP regulation	**2.35**	−0.66
T Cell Receptor Signaling Pathway	**2.33**	1.27
p38 MAPK Signaling Pathway (BioCarta)	**2.27**	−1.81
B Cell Receptor Signaling Pathway	**2.24**	0.20
Serotonin Receptor 4/6/7 NR3C signaling	**2.21**	−0.59
IL-5 Signaling Pathway	**2.07**	−0.69
G1 to S cell cycle control	**2.04**	−2.49
Cell cycle	**1.98**	NaN
TCA Cycle	**1.96**	NaN
Type II interferon signaling (IFNG)	**1.96**	−0.07
DNA damage response	**1.95**	−1.08
GPCRs, Class B Secretin-like	0.17	**2.95**
Inflammatory Response Pathway	0.13	**2.30**
Cholesterol Biosynthesis	−0.99	**1.91**

Pathway analysis is based on a comparison between recurrent and non-recurrent prostate cancer. Only significant pathways with a Z-score >1.9 in at least one of the two analyses are included. Significant Z-scores are depicted in bold. A NaN value commonly occurs when none of the genes in the pathway is present in the dataset.

Pathway analysis of the published data identified 14 significantly changed pathways (Table 5). Several of those indicated pathways with a Z-score higher than 1.9 were expected to be involved in prostate cancer recurrence, including the “Angiogenesis”, “G1 to S cell cycle control”, “Cell cycle”, and “DNA damage response” pathway. After standardized processing, PathVisio results indicated three pathways, the “GPCRs, Class B Secretin-like”, “Inflammatory Response Pathway” and “Cholesterol Biosynthesis” pathway, as being significantly dysregulated in disease relapse.

PathVisio analysis of the dataset by Best *et al.* [16] investigating differences in gene expression profiles of androgen-dependent and androgen-independent prostate cancer unraveled 15 significant pathways probably playing an essential role in prostate cancer progression to a more aggressive, androgen-independent type. The results of pathway analysis of the reprocessed data and the published data are shown in Table 6.

**Table 6:** PathVisio results of significant pathways found in the dataset by Best *et al.* comparing processed data provided by ArrayExpress with the reprocessed data after quality control.

Pathway	Z Score (ArrayExpress)	Z Score (Standardized processing)
*Cytoplasmic Ribosomal Proteins*	***9.59***	***6.46***
Catalytic cycle of mammalian FMOs	**3.37**	0.44
*Hypertrophy Model*	***2.86***	***2.88***
*Electron Transport Chain*	***2.72***	***2.13***
IL-1 Signaling Pathway	**2.41**	−0.41
Focal Adhesion	**1.99**	1.00
Nifedipine	**1.97**	0.69
Complement and Coagulation Cascades KEGG	**1.90**	1.20
Selenium metabolism/Selenoproteins	1.80	**2.05**
TGF-beta Receptor Signaling Pathway	1.30	**2.88**
ErbB signaling pathway	1.02	**1.95**
DNA damage response	0.82	**2.18**
Translation Factors	0.77	**2.48**
Blood Clotting Cascade	0.32	**2.36**
Oxidative Stress	0.11	**2.08**

Pathway analysis is based on a comparison between androgen-dependent and androgen-independent prostate cancer. Only significant pathways with a Z-score >1.9 in at least one of the two analyses are included. Significant Z-scores are depicted in bold; matches between the analyses are in italics.

Pathway analysis of the reprocessed data after QC detected 10 significantly changed biological pathways (Table 6). PathVisio results of the published data indicated 8 significantly changed pathways. Three overlapping pathways could be detected between the reprocessed data and the published data. These pathways included the “Cytoplasmic Ribosomal Proteins”, “Hypertrophy Model”, and “Electron Transport Chain” pathway, which appear to be linked with disease progression to androgen-independent prostate cancer.

## Discussion

An extensive literature search was performed in order to substantiate the pathway analysis results. Pathway analysis identified several signaling cascades and cellular processes that were overrepresented between the different datasets. These pathways and processes seemed to be characteristic for prostate cancer initiation and progression and could be assigned to three main biological processes, including cholesterol biosynthesis, epithelial-to-mesenchymal transition (EMT) involving epidermal growth factor receptor (EGFR) signaling, and an increased metabolic activity. Therefore, the final biological interpretation focused on these cellular processes, and their potential contribution to prostate cancer development.

### Cholesterol Biosynthesis

Several experimental and epidemiological studies provide strong evidence that the cholesterol biosynthesis plays a pivotal role in prostate cancer development and progression [22], [23].

Several studies have shown that cholesterol has the potential to accumulate in solid tumors and that cholesterol homeostasis gets disturbed in the prostate with advancing age and with the transition from a benign to a malignant state. Cholesterol accumulation in prostatic tumors likely occurs by several mechanisms, such as an increased cholesterol uptake from the circulation, loss of feedback regulation due to downregulation of low density lipoprotein receptors, and an upregulation of specific components of the mevalonate (cholesterol synthesis) pathway, like the 3-hydroxy-3-methylglutaryl-coenzyme A (HMG-CoA) reductase [23]. Therefore, elevated cholesterol levels in prostate cancer cells have been indicated to be the result from an aberrant regulation of the cholesterol metabolism [24]. These findings are in concordance with the pathway analysis results, as the “Cholesterol Biosynthesis” signaling pathway was found to be significantly altered during prostate cancer initiation and transformation of benign prostate tissue to primary prostate cancer (Table 2). Furthermore, the cholesterol metabolism appeared to be involved in prostate cancer recurrence (Table 5).

As cholesterol uptake and synthesis are linked with the cell cycle, the association between cholesterol, other lipogenic mechanisms and androgen action suggests the possibility that lipid products of these pathways play a role in androgenic stimulation of prostate cancer growth [23]. The lipid metabolism is a major target of androgenic signaling and is therefore tightly controlled by androgens in the normal prostate. Androgens are known to stimulate lipogenesis in prostate cancer cells directly by increasing the transcription of specific genes encoding lipogenic enzymes, such as the fatty acid synthase (FAS) and HMG-CoA reductase [22], [23]. For example, increased levels of FAS are associated with tumor formation and elevated levels of fatty acids have been identified to alter signaling processes at the plasma membrane. Furthermore, high FAS expression has been associated with an aggressive biological behavior, as the highest FAS levels are detected in androgen-independent bone metastasis [22], [25].

Recent studies investigating genes under transcriptional control of the androgen receptor revealed more than 300 androgen-responsive transcripts. The majority of these transcripts encode proteins that are involved in lipid metabolism. The androgen receptor is responsible for the recruitment of a group of transcription factors that drive the expression of the enzymes involved in lipid metabolism. These sterol response element binding proteins (SREBPs) consist of three related transcription factors, SREBP-1a, SREBP-1c, and SREBP-2 that have been indicated as critical regulators of androgen-regulated lipogenesis. SREBP-1c has been identified as being primarily responsible for the transcription of fatty acid biosynthesis genes, such as FAS, while SREBP-2 regulates genes of the cholesterol synthesis pathway, such as HMG-CoA reductase or farnesyl diphosphate synthase [22], [25]. Pathway analysis results provide additional verification of these findings, as the “Androgen Receptor Signaling Pathway” and “Fatty Acid Biosynthesis” appeared to be significantly altered during prostate cancer initiation (Table 5). Furthermore, the “Androgen Receptor Signaling Pathway” seemed to be significantly affected during the transition from primary to more aggressive, metastatic prostate cancer (Table 3). Interestingly, PathVisio analysis comparing androgen-dependent with androgen-independent prostate cancer did not detect the “Androgen Receptor Signaling Pathway” as significantly dysregulated (Table 5).

It has been indicated that the strictly coordinated expression and feedback regulation by this transcription factor family is frequently lost in prostate cancer. The elevated expression of a wide variety of genes involved in lipid metabolism supports an essential role of cholesterol synthesis in prostate cancer, but the underlying mechanisms of the uncontrolled activation of SREBPs remain widely unknown [24], [25].

Numerous signaling proteins have been identified to associate with plasma membrane lipid rafts, including the EGFR, the AR, heterotrimeric G-protein subunits, the T-cell receptor, as well as the interleukin-6 (IL-6) receptor. Signaling through the PI3K/Akt phosphorylation cascade has been demonstrated to be a frequent event in prostate tumors that harbor the inactivated lipid phosphatase tumor suppressor gene *PTEN*. It has been indicated that increased signaling through the *PI3K/Akt* signaling pathway, as a consequence of the loss of functional *PTEN*, is able to drive tumor progression. The EGFR leads to the activation of the *PI3K/Akt* pathway and therefore serves as mediator of solid tumor growth [22], [24].

Several of the indicated signaling proteins were also found in the pathway analysis results. The EGFR, AR and T-cell receptor, as well as several classes of G-protein coupled receptors appeared to be significantly altered during prostate cancer initiation (Table 4), the transition from primary to metastatic prostate cancer (Table 3), and in case of disease recurrence (Table 5).

To conclude, pathway analysis confirmed the results of several recent studies that identified cholesterol as playing a promotional role in prostate cancer. PathVisio analysis indicated a dysregulated cholesterol biosynthesis pathway as essential mechanism in prostate cancer initiation and progression to a more aggressive, metastasizing cancer type. Furthermore, cholesterol appeared to be an important element controlling signaling events in prostate cancer cells. It is suggested that the dysregulation of enzymes involved in cholesterol biosynthesis and metabolism may result in increased cholesterol levels in tumor cells. The destabilized cholesterol equilibrium may influence the transition from a coordinated process of cell proliferation and death to a severely altered condition, resulting in uncontrolled growth and progression to androgen-independent prostate cancer [22], [23].

### Epithelial-to-mesenchymal Transition

Pathway analysis revealed several signaling cascades, such as the EGFR1/ErbB-, TGF-β-, Wnt-, Delta-Notch- and TNF-α/NF-κB signaling pathways, that can be assigned to a main process known as epithelial-to-mesenchymal transition. EMT is a key event during embryonic development that is required for morphogenetic movements during the reorganization of the embryonic germ layers. The process of EMT has been well documented in cell lines and mouse experiments, but its clinical relevance remains controversial [26]. However, several recent studies provide evidence that EMT is linked to cancer progression, invasion, and metastasis [27]. The process of EMT has therefore been proposed as hallmark of carcinoma progression towards a dedifferentiated and more malignant state [28], [29].

Cells that undergo EMT are characterized by transient structural changes resulting in loss of polarity and contact with neighboring cells [30]. EMT is characterized by the repression of E-cadherin expression, and increased cell motility. The loss of E-cadherin has been demonstrated as a marker of EMT and seems to correlate with dedifferentiation, local invasiveness, and metastasis formation of prostate cancer cells [26], [30], [31].

Several oncogenic pathways like the Wnt-, TGF-β-, Hedgehog-, TNF-α/NF-κB-, EGFR-, and Notch-signaling pathway are supposed to initiate EMT [27], [29]. For example, the transforming growth factor-β (TGF-β) has been characterized as potent EMT inducer in normal embryonic development, and during cancer progression. TGF-β induces EMT and causes the dissolution of cell-junction complexes. Furthermore, EMT coordinates the cooperation between oncogenic Ras and receptor tyrosine kinases to induce downstream Raf/MAPK signaling that is strongly associated with prostate tumor progression and poor clinical prognosis [27].

Overexpression of the EGFR family has been associated with disease progression of numerous malignancies including prostate cancer. In prostatic tumors, EGFR has been indicated to initiate EMT in cooperation with TGF-β, and enhances the invasion of prostate cancer cells. In the presence of androgens, endogenous and ectopically expressed AR directly associates with EGFR and decreases the activation of downstream PI3K signaling leading to cancer cell growth and survival. EGFR may also sensitize prostate cancer cells to low levels of androgens by enhancing co-activator binding and transcriptional activation of endogenous and ectopically expressed AR. Therefore, the observed cross-talk between the AR and EGFR axes leads to the assumption that EGFR-induced EMT and androgen-independence could occur simultaneously in prostatic tumor cells [26].

Another important pathway playing an essential role in the development and progression of prostate cancer is the HIV-I NEF pathway. This pathway comprises the tumor necrosis factor- (TNF) and FAS receptor signaling pathways and seems to be particularly dysregulated in androgen-independent metastatic prostate cancer compared with localized primary prostatic tumors [27]. These findings are only partly consistent with the pathway analysis results. As expected, the FAS receptor signaling pathway appeared to be significantly affected in metastatic prostate cancer (Table 3) and during disease recurrence (Table 5), but neither the Fas receptor- nor the TNF-α signaling pathway were found to be significantly dysregulated in androgen-independent prostate cancer (Table 6).

According to literature, especially the TNF branch of the HIV-I NEF pathway seems to be of high importance and consists of the activation of nuclear factor-κB (NF-κB) by TNF-α [32]. The binding of TNF-α to its receptor leads to the dissociation of the inhibitory protein SODD and recruitment of an adapter protein, known as TRADD. TRADD binds to additional adapter proteins TRAF2 and RIP1 causing the recruitment and activation of the IKK complex. This activation in turn results in the phosphorylation and dissociation of IκBα from the NF-κB heterodimer and translocation of the active heterodimer to the nucleus. Translocation initiates the transcription of target survival pathway genes including TRAF proteins and inhibitors of apoptosis. It has been confirmed that this pathway is highly dysregulated in androgen-independent metastasis [32].

In conclusion, pathway analysis indicated several significant signaling cascades that are in concordance with findings in the literature, such as the TGF-β, TNF-α/NF-κB-, and EGFR-signaling cascade. The interplay of several of such extracellular signaling molecules, growth factors, and transcription factors has been suggested to induce EMT and possesses the potential to serve as EMT marker [32]. Pathway analysis could demonstrate and confirm that the Wnt-, Delta-Notch- and EGFR1 signaling pathway were significantly dysregulated during prostate cancer initiation and formation of a clinically localized, primary prostate tumor (Table 2 and 4), as well as during disease progression to a more aggressive, metastatic phenotype (Table 3). Furthermore, the Delta-Notch and TGF-β signaling pathway could be confirmed as essential initiators of metastasis (Table 3).

### Increased Metabolic Activity in Prostate Carcinogenesis

Pathway analysis results led to the assumption that an altered metabolic activity might be involved in prostate carcinogenesis, as the analysis detected pathways like “mRNA processing”, a posttranscriptional modification involving enzymatic activity, the “Electron Transport Chain” (ETC), and “Oxidative phosphorylation” that are actively involved in metabolism. Those pathways were found to play an essential role during prostate cancer initiation and transition from a benign to a malignant state (Table 4). The ETC also appeared to be essential during disease progression to a more aggressive tumor type (Table 6).

Several recent studies provide evidence for a promotive role of an increased metabolic activity in prostate cancer tumor growth. It has been shown that a disrupted respiratory chain activity resulting from mutations in mitochondrial DNA (mtDNA) in prostate cancer cells leads to overproduction of ROS contributing to tumor growth [33]–[35].

Normal prostate epithelial cells are unique cells that accumulate high concentrations of zinc, which is able to inhibit enzymes involved in the citrate metabolism through the Krebs cycle. A malignant transformation of the prostate is associated with an early metabolic switch, causing decreased zinc accumulation and increased citrate oxidation by activating the enzyme m-aconitase [33]. The associated downregulation of the zinc uptake transporter by transforming glandular epithelial cells serves as an early marker of metabolic alteration. The metabolic transformation from energy inefficient benign cells to energy efficient tumor cells implies an increased ETC activity, increased consumption of oxygen, and excessive production of ROS leading to oxidative stress which induces accumulating mutations in the vulnerable mtDNA [33], [34].Certain mtDNA mutations may cause alterations of the electron transport components of the ETC that comprise the normal electron flow [34]. For instance, mutations in the cytochrome oxidase subunit I gene of the ECT have been shown to initiate prostate cancer [35].

The ROS induced mitochondrial dysfunction is subsequently able to activate nuclear genes and signaling pathways involved in tumor initiation and progression. For example, ROS are able to induce pathways, like the TNF-α/NF-κB- and PI3K signaling pathway that are involved in increased hypoxia-inducible factor α (HIFα) expression and that activate genes playing an essential role in angiogenesis and tumor metastasis, thereby contributing to tumor growth. Also pathway analysis results indicated the TNF-α/NF-κB signaling pathway as being significantly dysregulated in prostate carcinoma formation (Table 4). In addition, ROS mediated disruption of mitochondrial functions has been demonstrated to initiate the calcium-dependent protein kinase C pathway. This pathway activates several downstream genes, such as cathepsin L, that play a role in prostate tumor invasiveness [33]. However, pathway analysis did not detect this signaling cascade as significantly dysregulated.

Pathway analysis was able to confirm the results of several recent studies and identified an increased metabolic activity as a key process of prostate cancer initiation and progression. A metabolic switch of prostatic cells has been indicated as key event during the transformation of benign epithelial cells into malignant cells. Furthermore, mitochondrial dysregulation as a consequence of elevated ROS production has been shown to play an essential role in prostate tumor growth and metastasis [33]–[35].

When comparing the results of the pathway analyses of the data processed by the standardized procedure and the data from ArrayExpress, the level of correspondence differs. For some datasets, large differences are observed, which could be caused by differences in any of the analytical steps, including (i) the removal of some of the arrays in the QC phase, (ii) the preprocessing and normalization methods applied, or (iii) the annotation of the reporters. We observed that none of the individual analytical steps in the standardized procedure consistently exerts the strongest effect on pathway analysis results (results not shown). This also essentially depends on the original quality of the dataset and the methods originally used. The paper shows however, that a systematic analysis of existing datasets using a standardized approach is feasible and leads to meaningful and verifiable results, thereby stimulating reuse of already available datasets and reducing cost. Furthermore it demonstrates that in several publicly available datasets, arrays of low quality are still present. Using a pathway approach may, however, make study outcome more robust to individual variations between datasets.

### Conclusion

The application of pathway analysis using PathVisio on multiple datasets led to the identification of several signaling pathways and cellular processes that play an important role in prostate cancer development and that subsequently were assigned to three main biological processes, including cholesterol biosynthesis, epithelial-to-mesenchymal transition and an increased metabolic activity. These results were confirmed with findings in the literature. It has been demonstrated that the indicated cellular processes are key contributors to prostate carcinogenesis and metastasis. An altered cholesterol metabolism has been shown to initiate prostate cancer and to promote the transition from a benign into a malignant state. Preclinical studies indicated that the process of EMT was considered as a hallmark of prostate cancer progression and metastasis, while an increased metabolic activity has been demonstrated to contribute to prostate tumor growth and invasiveness as a consequence of ROS-induced mitochondrial dysregulation. These processes may deliver candidates for new biomarkers, and novel targets for therapeutic regimes. Identifying the most commonly altered pathways in both primary and metastatic cancer could lead to building more detailed and realistic, disease-specific maps. Super-imposing expression data may help discriminating treated versus non-treated patients or even improve our understanding of a drug’s mechanism of action or resistance.

In conclusion, we have demonstrated that the application of a standardized bioinformatics workflow, including QC, statistical analysis and pathway analysis, to publicly available datasets, serves as a powerful and cost and time effective approach to reveal the most relevant biological mechanisms underpinning prostate cancer development and progression. Being a generic approach, it can be similarly applied to datasets related to any other disease or condition of interest.

## Supporting Information

Appendix S1 Detailed description of microarray data analysis.
(DOCX)

Appendix S2 Supplemental data.
(DOCX)
