# Supplementary material for: Molecular Pathways Involved in Prostate Carcinogenesis: Insights from Public Microarray Datasets
Source: PLoS One. 2012 Nov 20;7(11):e49831. doi: 10.1371/journal.pone.0049831 (PMC3502280; doi:10.1371/journal.pone.0049831)
Supplement: Appendix S1 — Detailed description of microarray data analysis. (DOCX) [file pone.0049831.s001.docx]

**Appendix S1: Detailed description of microarray data analysis**

***Microarray data analysis and quality control criteria***

The process of microarray data analysis can be summarized in four main steps, including raw data QC, data pre-processing, evaluation of the effect of normalization, and biological knowledge extraction. Essentially, the application of QC to microarray datasets is subdivided into four quality indicators: sample quality, hybridization quality, signal comparability, and array correlation.

In a first step, raw data CEL files of each microarray dataset were imported in R (version 2.13.0) using the ReadAffy function of the affy package of Bioconductor 2.8. Sample quality was evaluated by the assessment of sample preparation and RNA controls. Sample preparation controls include probe sets from several B. subtilis genes (lys, phe, thr, dap), which are spiked at the beginning of the chip processing, and provide a useful tool to determine the overall success of the target preparation steps. These poly-A controls Lys, Phe, Thr and Dap should be called present at increasing intensity to confirm that no bias has occurred during the retro-transcription between highly expressed and low expressed genes. A probe set is called present if the perfect match (PM) value is significantly larger than the mismatch (MM). The inclusion of RNA quality controls provides a useful approach to assess overall RNA sample, multiplication, and assay quality. RNA quality controls serve as indicator for the 3´/5´ and 3´/M ratio of housekeeping genes, such as β-actin (beta-actin) or GAPDH (Glyceraldehyde 3-phosphate dehydrogenase), which are expressed in most cell types. Three probe sets are designed to three regions of these genes (3´, 5´ and M (middle)) giving an indication of the integrity of the starting RNA and efficiency of the first strand cDNA synthesis. In general, a higher signal for the 3´ probe set than for the M and 5´ probe sets is expected, because of the direction of cDNA synthesis. The signal values of the 3´ probe sets for β-actin and GAPDH are compared to the signal values of the corresponding 5´ probe sets. For an array of good quality, Affymetrix recommends that the 3´/5´ ratio of β-actin should not exceed 3, while the 3´/5´ ratio of GAPDH should not exceed 1.25.

Overall RNA quality was assessed by RNA degradation plots. This plot shows the average intensity of each probe across all probe sets, ordered from the 5´ to the 3´ end. It is expected that probe intensities are lower at the 5´ end of a probe set when compared to the 3´end as RNA degradation starts from the 5´end of a molecule. RNA which is too degraded will show a very high slope from 5´ to 3´. Thus, the standardized slope of the RNA degradation plot serves as quantitative indicator of the RNA degradation.

Hybridization quality was assessed using hybridization spike-in controls, determining the percent present values, evaluating positive and negative control distributions, as well as defining background intensities. A concentration range of hybridization controls BioB, BioC, BioD and CreX is spiked to the microarray prior to the labeling process. Subsequently, the intensity patterns of the hybridization controls are measured to evaluate sample hybridization efficiency, expecting a pattern corresponding to the target concentration. Other patterns are regarded as a sign of poor hybridization.

The determination of the percent present values is used to determine the percentage of present probe sets relative to the total number of probe sets on the array. These percentages should range from 30 to 50 % and should be comparable across arrays. The subsequent assessment of positive and negative control distributions leads to the creation of cut-off values for arrays to be kept. Furthermore, the background intensity of each array is defined from the MM probes value. It is required that average, minimum and maximum background are comparable between different arrays, as a significantly higher or lower background value is indicative of poor quality.

Signal comparability and biases were assessed by using several visualizations, such as a plot of log scale factors, box plots and density histograms of log-intensities, MA plots and spatial images. MA plots allow pairwise comparison of the log-intensity of each array to a reference array and identification of intensity-dependent biases. The y-axis of the MA-plot shows the log-ratio intensity of one array to the reference median array, which is called M (minus). The x-axis indicates the average log-intensity of both arrays, which is called A (add). It is expected that the probe levels do not differ systematically within a group of replicates, so that the MA-plot is centered on the y-axis (y=0) from low to high intensities. Furthermore, normalization is expected to correct for intensity-dependent biases. MA-plots are used before and after normalization to verify the efficiency of this correction.

Spatial images are artificial visualizations of an array that are created to detect spatial trends or biases on an array. Upon scanning, a scale factor is applied to each array of a dataset in order to equalize the arrays` mean intensities. A dataset of good quality should have comparable scale factors with limited variation. As recommended by Affymetrix, the scale factors should be within 3-fold of one another for a dataset. Box plots and density histograms of log-intensities before and after normalization are used to check the normalization step. After normalization, the distribution of the normalized probe set log-intensities is expected to be more comparable if not identical between the different arrays. Probe sets homogeneity is determined by Normalized Unscaled Standard Error (NUSE) and Relative Log Expression (RLE) plots. The NUSE plot represents normalized standard error estimates from the Probe-Level Model (PLM) fit which computes expression measures on a probe set by probe set basis. The standard error estimates are normalized so that the median standard error for each probe set across all arrays is equal to 1. A box plot of NUSE values is drawn for each array in a dataset and allows checking whether all distributions are centered near 1 and whether an array shows a globally higher spread of the NUSE distribution than the other arrays. Typically, a box centered above 1.1 is indicative of an array with quality problems. The RLE values are computed by calculating the ratio between the expression of a probe set and the median expression of this probe set across all arrays of a dataset. It is assumed that most probe sets are not changed across the arrays, so that ratios around 0 on a log scale are expected. The RLE box plots should then be centered near 0 and should show a comparable spread. Other patterns are indicative of low quality arrays.

Correlation between arrays was evaluated by the creation of correlation plots, the performance of hierarchical clustering of arrays, and principal component analysis (PCA). In correlation plots, a correlation coefficient for each pair of arrays in the dataset is qualitatively presented as a colored matrix. The degree of correlation is measured between -1 and 1, with 1 being considered a perfect correlation and 0 representing no correlation. Therefore, low coefficients are indicative of important differences between array intensities. Hierarchical clustering analysis is performed in two steps: first, the distances between all pairs of arrays are computed and second, a tree is created from these distances by repeatedly grouping those (groups of) arrays together that are closest to each other. PCA plots the information contained in the dataset in a reduced number of dimensions. Clustering and PCA plots enable the assessment to what extent arrays resemble each other, and whether this corresponds to the known resemblances of the samples.

Application of several of the plots and quality indicators after normalization further assisted in determining the efficacy of the normalization method and in assessing the quality of the dataset. The GC Robust Multi-array Average (GCRMA) method served as normalization method during the microarray QC procedure and updated gene annotations from Brainarray (http://brainarray.mbni.med.umich.edu) were used. GCRMA is an adapted form of RMA that is able to use sequence-specific probe affinities of the Affymetrix GeneChip probes to obtain more accurate gene expression values. Starting with probe-level data of several GeneChips, the PM values are background-corrected, normalized and summarized resulting in a single expression measure per probe set and per array. The background correction step used in GCRMA is designed to account for possible background noise and non-specific binding. Normalization is necessary to be able to compare multiple arrays with each other and to analyze them together.

It needs to be emphasized that the application of QC is a cyclic process that needs to be re- performed until the QC gives positive results: After indicating and removing low quality data, QC is performed again with the remaining arrays to verify dataset quality. Furthermore, by re-computing the QC step smaller aberrations can be detected that had been obscured before by larger ones.

In a final step, the normalized expression data were exported to a text file for each dataset. All plots and images created by the microarray QC workflow were saved as .png files in the respective working directory.

***Statistical analysis***

Statistical analysis was applied to the obtained processed data of each dataset before and after QC, and to the processed data as provided on ArrayExpress.

Statistical analysis was performed using the R package limma of the Bioconductor project. A “Group” factor was assigned to contain the experimental grouping of all arrays in each dataset and a design matrix was set up with rows corresponding to sample names and columns corresponding to experimental groups. Using the lmFit function in the limma package a linear model was fitted to the expression data for each probe set in the series of arrays. The expression data need to be log-transformed prior to fitting a linear model. Subsequently, the empirical Bayes method (eBayes function) for differential expression was used to estimate eBayes values and to perform a moderated t-test. In case that more than two different groups are investigated within a dataset, the makeContrasts function is used to compare each group with the other groups. The pairwise comparisons are depicted in a table using the topTable function of the limma package. This table contains the fold changes on a logarithmic scale (logFC), the fold changes (FC) themselves, the average expression (AveExpr) value, the t- and B-statistics, and the calculated (P.Value) as well as adjusted p-value (adj.P.Val.) per gene. Furthermore, the external gene ID and gene description per probe are included in the table. The table was stored as tab-delimited text file for the processed data of each dataset, and for the processed data from ArrayExpress.

***Pathway analysis***

After the QC procedure, data of sufficient quality were obtained. For the final biological knowledge extraction, pathway analysis was performed using PathVisio with WikiPathways content. The analysis was done after applying statistics to the processed data of each dataset, and to the processed data downloaded from ArrayExpress. The tab-delimited text file containing the average normalized expression values, fold changes, p-values and adjusted p-values of all measured genes per dataset, which was obtained from the statistical analysis, was imported into PathVisio. A color gradient based on the log fold changes was used to visualize and determine differences in gene expression values between the experimental groups in a dataset. Down-regulated genes were depicted in blue, while up-regulated genes were shown in yellow. Unchanged genes were presented in grey. Furthermore, the degree of significance of the change as indicated by the calculated p-value was depicted in red. A p-value smaller than 0.001 ([P.Value] < 0.001) was shown in dark red, while a p-value smaller than 0.05 ([P.Value] < 0.05) was depicted in light red. A p-value smaller than 0.01 ([P.Value] < 0.01) served as intermediate red-colored p-value. Using the statistics function in PathVisio, a list of pathways was generated ranking the pathways based on their calculated Z-score. A p-value smaller than 0.05 ([P.Value] < 0.05) served as criterion to compute a pathway`s overrepresentation of significantly changed genes between experimental groups. All pathways with a Z-score higher than 1.9 (Z-score > 1.9) were included in the biological interpretation and knowledge extraction, resembling a significance level of 0.05.
