# Supplementary material for: Molecular Pathways Involved in Prostate Carcinogenesis: Insights from Public Microarray Datasets
Source: PLoS One. 2012 Nov 20;7(11):e49831. doi: 10.1371/journal.pone.0049831 (PMC3502280; doi:10.1371/journal.pone.0049831)
Supplement: Appendix S2 — Supplemental data. (DOCX) [file pone.0049831.s002.docx]

**Appendix S2: Supplemental data**

***Quality control results***

All quality control results can be downloaded from:

http://www.bigcat.unimaas.nl/public/publications/thesis/Baetke/
